# The Impact of Retinal Configuration on the Protein–Chromophore Interactions in Bistable Jumping Spider Rhodopsin-1

**DOI:** 10.3390/molecules27010071

**Published:** 2021-12-23

**Authors:** Jonathan R. Church, Jógvan Magnus Haugaard Olsen, Igor Schapiro

**Affiliations:** 1 Fritz Haber Center for Molecular Dynamics Research, Institute of Chemistry, The Hebrew University of Jerusalem, Jerusalem 9190401, Israel; jonathan.church@mail.huji.ac.il; 2 DTU Chemistry, Technical University of Denmark, DK-2800 Kongens Lyngby, Denmark; jmho@kemi.dtu.dk

**Keywords:** rhodopsins, bistable, jumping spider, QM/MM, spectral tuning

## Abstract

Bistable rhodopsins have two stable forms that can be interconverted by light. Due to their ability to act as photoswitches, these proteins are considered as ideal candidates for applications such as optogenetics. In this work, we analyze a recently crystalized bistable rhodopsin, namely the jumping spider rhodopsin-1 (JSR1). This rhodopsin exhibits identical absorption maxima for the parent and the photoproduct form, which impedes its broad application. We performed hybrid QM/MM simulations to study three isomers of the retinal chromophore: the 9-*cis*, 11-*cis* and all-*trans* configurations. The main aim was to gain insight into the specific interactions of each isomer and their impact on the absorption maximum in JSR1. The absorption spectra were computed using sampled snapshots from QM/MM molecular dynamics trajectories and compared to their experimental counterparts. The chromophore–protein interactions were analyzed by visualizing the electrostatic potential of the protein and projecting it onto the chromophore. It was found that the distance between a nearby tyrosine (Y126) residue plays a larger role in the predicted absorption maximum than the primary counterion (E194). Geometric differences between the isomers were also noted, including a structural change in the polyene chain of the chromophore, as well as changes in the nearby hydrogen bonding network.

## 1. Introduction

**Figure 1:**
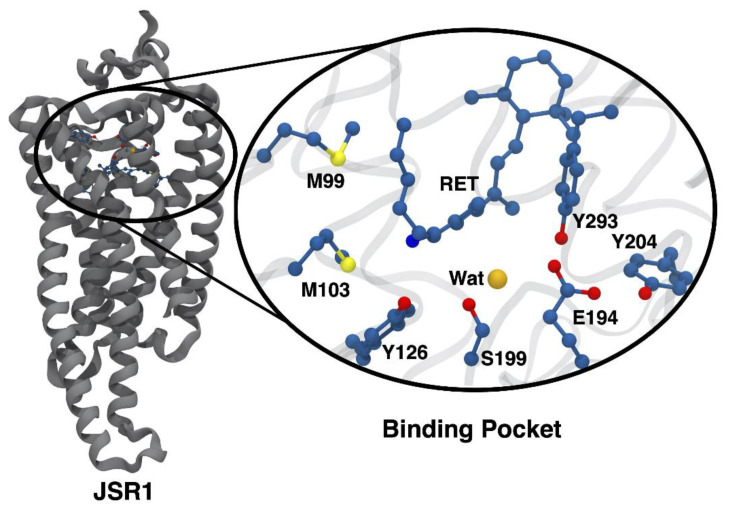
Crystal structure of jumping spider rhodopsin-1 (JSR1), containing the 9-*cis* form of retinal, taken from the Protein Data Bank (PDB ID: 6I9K) [7]. Several key residues in the retinal-binding pocket are also shown. These include the counterion E194 and a crystallographic water thought to be important to the hydrogen bonding network linking the RPSB.

Rhodopsins constitute a family of photoreceptor proteins that share a common secondary structure of seven transmembrane helices [1]. These proteins gain their light sensitivity through an attached retinal (RET) chromophore that is covalently bound to the protein through a lysine residue. Together, lysine and RET form a retinal protonated Schiff base (RPSB) at their link. Rhodopsins can be further grouped into either monostable or bistable rhodopsins, depending on the number of thermally stable intermediates in the photocycle of the protein. In the case of monostable rhodopsins, such as those found in bovine rhodopsin, the RPSB deprotonates along the photocycle. After the deprotonation event and a series of conformational changes of the protein, the Schiff base bond undergoes hydrolysis that ultimately leads to the release of the chromophore and bleaching of the sample [2]. In contrast, the photoproduct of bistable rhodopsins is thermally stable, yielding a long-lived product that does not undergo bleaching. This characteristic of bistable rhodopsins is thought to be due to the RPSB remaining protonated throughout the photocycle [3,4]. This ability also elevates bistable rhodopsins as interesting candidates for optogenetics and other biotechnological applications, due to their ability to act as a photoswitch between the parent form and photoproduct [5]. Jumping spider rhodopsin-1 (JSR1) is such a bistable rhodopsin, first discovered within the jumping spider *Hasarius adansoni* by Nagata and coworkers in 2012 (Figure 1) [6]. It also has the advantage of being a type II or animal rhodopsin, which is supposedly better suited for optogenetics in mammalian cells.

**Figure 2:**
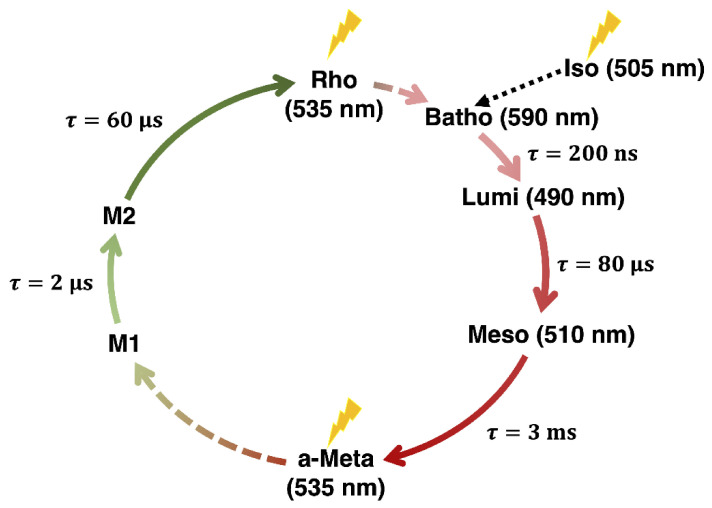
Photocycle of JSR1 showing the conversion between the Rho (11-*cis*) and a-Meta (all-*trans*) states, as well as conversion of Iso (9-*cis*) to the Batho intermediate.

The photocycle of JSR1 consists of a series of intermediates between the parent form (Rho) and an acid-Meta (a-Meta) photoproduct (Figure 2) [2,3,5,8]. The nomenclature of the intermediates is adapted from bovine rhodopsin [9]. Both Rho and a-Meta can undergo photoisomerization. Rho isomerizes from the 11-*cis* isomer of retinal (RET) chromophore to its all-*trans* form, while a-Meta undergoes the reverse reaction. However, the Rho and a-Meta forms have the same experimental absorption maxima at 535 nm [2,10]. The coinciding absorption maxima make the spectroscopic characterization difficult, because the yield of each reaction is not unity, and therefore, a mixture of these two forms is obtained [2]. Ehrenberg et al. characterized the photocycle of JSR1 using IR and UV/Vis spectroscopy of JSR1 reconstituted with the 9-*cis* RPSB [2]. This isomer exhibits a different absorption maximum from the 11-*cis* and all-*trans* chromophores, namely 505 nm. Following illumination, 9-*cis* isomerized predominantly to the Batho intermediate, which contains the all-*trans* retinal chromophore [2]. Ehrenberg et al. found that Rho-to-a-Meta conversion passes through three intermediates: Batho (τ = 200 ns, $\lambda_{\max}$ = 590 nm), Lumi (τ = 80 μs, $\lambda_{\max}$ = 490 nm) and Meso (τ = 3 ms, $\lambda_{\max}$ = 510 nm). After forming a-Meta, an additional photon can regenerate Rho, which is thought to be linked by two intermediates whose maximum absorbances were not directly observed, M1 (τ = 2 μs) and M2 (τ = 60 μs).

Initially, Nagata et al. suggested that E194 is the counterion in JSR1, responsible for stabilization of the RPSB of both the parent and the photoproduct forms [10]. Unlike monostable rhodopsins, where the RPSB has direct interactions with the nearby counterion, in JSR1 the E194 counterion is too far for direct interactions with the RPSB (Figure 1). The long separation between the counterion and RPSB implies that the charge of the chromophore is stabilized through indirect interactions with E194, such as an extended hydrogen bonding network, although the exact mechanism is still unknown [10]. A mutational analysis in combination with pK_a_ studies by Nagata et al. suggested that the mechanism for the interaction between the RPSB and the local environment of the binding pocket is different for both the photoproduct and parent forms [10]. The serine in position 199 was targeted with several mutations, including S199F and S199A. Changes in this position were found to affect the pK_a_ and absorption maximum of the parent form to a much greater degree than that of the photoproduct. This suggests that there are different stabilizing mechanisms for the parent and photoproduct forms of JSR1. Similarly, mutations such as E194Q and E194D showed drastic changes to the absorption maximum for each isomer, as well as the pK_a_ of the protonated Schiff base, suggesting that this residue plays an important role in both the parent and photoproduct [10]. 

Each isomer of retinal chromophore also exhibits unique FTIR and Raman spectra inside the protein environment that can be used to experimentally identify which isomer is bound to the protein. Structural information of the chromophore itself can also be obtained from Raman data, where hydrogen-out-of-plane (HOOP) modes are shown to be low intensity for a-Meta relative to Rho, suggesting structural changes in the polyene chain of the chromophore upon formation of the photoproduct [2]. In this work, we used the recently crystallized structure containing the 9-*cis* chromophore to model and investigate the effect that the 9-*cis*, 11-*cis* and all-*trans* isomers have on the local environment of the binding pocket in JSR1 using a combination of MM and hybrid QM/MM simulations. 

## 2. Computational Methods

### 2.1. Model Generation

The crystal structure reported by Varma et al. (PDB ID: 6I9K) served as a basis for this study [7]. The 6I9K crystal structure was comprised of JSR1 with a bonded 9-*cis* RPSB. Missing loops were added using the Modeller program [11]. Due to the lack of crystal structures containing the 11-*cis* and all-*trans* isomers, the crystal structures of squid rhodopsin (SR) were used (PDB IDs: 2Z73 [12] and 3AYM [13]) as a template for the orientation of the retinal chromophore. SR was already used to refine the crystal structure by Varma et al. due to their similar structural features, including the hydrogen bonding network [7]. In this work, the crystal structures of SR were used due to the structural similarity between JSR1 and SR but, more importantly, due to the similarity in the binding pocket [7]. The peptide backbone of each these crystal structures was first aligned to that of 6I9K, and the structures were generated by replacing the original 9-*cis* chromophore with the 11-*cis* and all-*trans* isomers. Each of the resulting structures were then embedded inside a bilayer of POPC lipid molecules using the CHARMM-GUI website [14]. The protonation states of each titratable residue were further checked and assigned using the PROPKA3.0 program [15,16].

### 2.2. Retinal Parameterization

The parameters for each isomer of the retinal chromophore were derived using the mdgx program found in AmberTools 16 [17]. The bond, angle and dihedral parameters, as well as the atomic RESP charges for each isomer, were produced using gas-phase trajectories. Each isomer of the retinal chromophore was first optimized in the gas phase using HF/6-31G*, the lysine link, including the backbone atoms, were kept, and the severed peptide bonds were capped using hydrogens. Next, 30 initial conditions for each conformer in the gas phase were generated using normal mode sampling, where the energy of each normal mode was sampled using a Boltzmann distribution of energies at 300 K. The trajectories were then propagated using the GAMESS-US (2018, R3) program at the HF/6-31G* level of theory for 20 fs, each using a timestep of 0.5 fs [18]. Snapshots were then generated using the resulting trajectory swarm every 1 fs, and a RESP fitting was performed using the mdgx program in tandem with ORCA 4.2.0 at the HF/6-31G* level of theory [19,20]. To account for the ability of the chromophore to isomerize between the *cis* and all-*trans* isomers, we fit a set of parameters using the snapshots of both 9-*cis* and all-*trans*, and another set using those of 11-*cis* and all-*trans*. This resulted in the fitting of 1260 snapshots for both the 11-*cis*/all-*trans* and 9-*cis*/all-*trans* parameter sets. During the fitting, the point charges of the lysine link were fixed to that of normal lysine, except for epsilon carbon and its hydrogens. A restraint weight of 0.005 kcal/mol was placed on the charges of all heavy atoms of the chromophore when performing the RESP fitting. Equivalent hydrogens were also restrained to have the same value in the charge fitting. The RESP charges were then used to fit the bond, angle and dihedral parameters at the B3LYP/cc-pVTZ level of theory. During these calculations the RIJCOSX approximation was used along with a D3 dispersion correction and a tight SCF convergence criterion. The force field parameters were then iterated by optimizing the original set of snapshots with the mdgx program, together with the Amber program, which generated new structures, and this process was repeated until the parameters converged. The final parameter sets are reported in the Supplementary Materials.

### 2.3. Classical and QM/MM Molecular Dynamics Simulations

The resulting structures from the CHARMM-GUI website were minimized and heated, and then, classical dynamics were performed for 380 ns using the Amber ff14SB force field for the protein and lipid14 for the POPC bilayer, along with our derived parameters for the retinal chromophores [21,22]. The 9-*cis*/all-*trans* parameter set was used for both the 9-*cis* and all-*trans* models. SHAKE was used to constrain the motion of the bonds involving hydrogen unless otherwise noted. First, each model was minimized using the classical force field for 100,000 steps. During the minimization, a restraint weight of 10 kcal/mol was placed on all atoms, including hydrogens. A thermal equilibration step was then performed by heating the models from 0 to 300 K at constant volume and temperature for 500 ps using a timestep of 1 fs with constraints placed on the heavy and light atoms, as outlined in the Supplementary Materials (Table S1). Next, six separate steps of equilibration were performed for 250 ps each at a constant pressure of 1 atm and temperature of 300 K while the restraints were slowly released (see Methodology). After releasing all restraints, an MD run was performed for 300 ns at constant pressure and temperature with a 2-fs timestep. Finally, SHAKE was removed from the protein, and an additional 80-ns equilibration step was performed with a timestep of 1 fs. To generate snapshots for spectra calculations, additional QM/MM MD trajectories were produced by continuing the aforementioned MM trajectories using the DFTB2+D method as implemented in Amber with an electrostatic embedding scheme. The QM/MM molecular dynamics were performed using the Amber package [17]. The QM region was defined as the retinal chromophore and lysine link cut between the C_β_-C_α_ bond and capped with a hydrogen atom to exclude the peptide backbone. The MM partition included the remaining protein, lipids, ions and waters that were treated with the classical ff14SB Amber force field using a 12-Å nonbonding cutoff [21]. DFTB was chosen to treat the QM region, because this method has been shown to yield structures in close agreement with those obtained using the B3LYP functional with medium-sized basis sets while remaining computationally feasible for trajectories in the nanosecond timescale [23,24,25]. These QM/MM trajectories were propagated for 1 ns with a timestep of 1 fs at 300 K using the Langevin thermostat and keeping the pressure at 1 atm. During these trajectories, both the QM and MM regions were fully relaxed, and SHAKE was not used to constraint the hydrogens of the protein. The resulting QM/MM trajectories were then used to generate snapshots for spectra generation of each model by taking a snapshot of the trajectory every 10 ps, resulting in 100 snapshots [26].

### 2.4. Spectra Generation

The excitation energies were determined with the time-dependent density functional theory (TD-DFT) using the CAM-B3LYP functional and cc-pVDZ basis [27,28]. The TD-DFT excitation energies of each snapshot were determined for the lowest 10 excited states using electrostatic embedding. The QM region consisted of the chromophore and the lysine sidechain cut between C_α_ and C_β_, which was capped with a hydrogen link atom. The TD-CAM-B3LYP calculations were performed using the Dalton program [29,30]. The protein, lipids, ions and solvent in the calculations were described using point charges from the ff14SB and lipid14 force fields, with a 50-Å cutoff from the RET residue. The 50-Å cutoff included the entire protein and a number of ions, waters and lipid molecules, as detailed in Table S2 of the Supplementary Materials and visualized in Figure S1. Each set of the resulting stick spectra were then broadened, assuming a Gaussian band shape with a width of 0.15 eV, and the final spectra were determined by averaging over the 100 conformations at each wavelength.

## 3. Results and Discussion

### 3.1. Structural Differences between the Isomers

**Figure 3:**
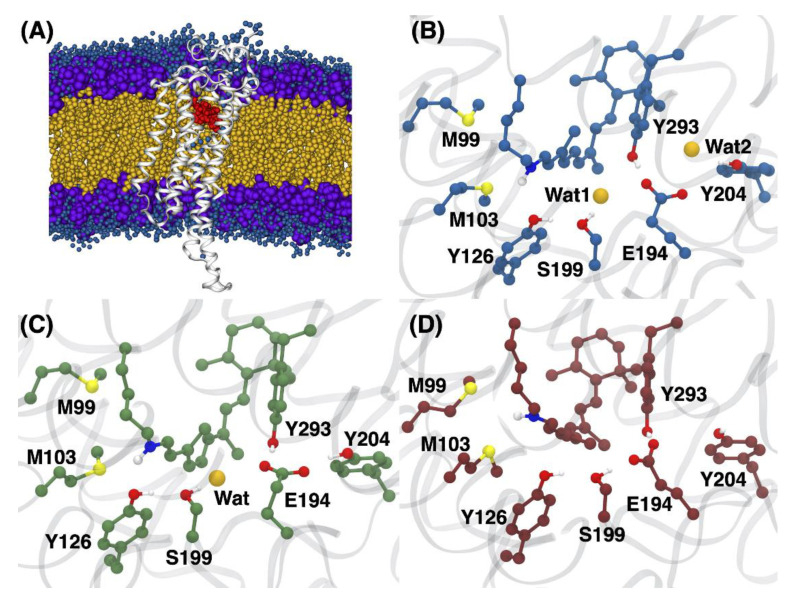
(**A**) JSR1 protein embedded in a membrane following the production procedure outlined in the methodology, along with the chromophore shown in red and nearby waters represented as blue spheres. (**B**–**D**) The 9-*cis*, 11-*cis* and all-*trans* RPSB, with key residues in the binding pocket.

The analysis of the resulting QM/MM MD trajectories for the 9-*cis*, 11-*cis* and all-*trans* RPSB isomers showed several structural differences in the arrangement of the binding pocket (Figure 3). The average structure of each isomer was calculated from the trajectories and visualized to analyze any differences caused by the configuration of the chromophore (Figure 3B,C). The 9-*cis* and 11-*cis* models were found to each contain a water linking E194 and S199 through hydrogen bonds (Figure 3B,C). This water has been suggested to participate in the hydrogen bonding network, linking the distant E194 to the RPSB for Rho of JSR1 [10]. In the case of the all-*trans* chromophore, the polyene chain of chromophore twists in such a way that a methyl group at C_13_ occupies the water position found in the 9-*cis* and 11-*cis* structures, leading to a loss of the water between E194 and S199. The loss of this water causes the direct interaction of the E194 counterion with that of S199 (Figure 3D). The change in the polyene chain is consistent with the Raman spectra analysis performed by Ehrenberg et al., who reported that the intensity of the HOOP modes belonging to the retinal chromophore change upon isomerization between the 11-*cis* and all-*trans* isomers, indicating a distorted polyene chain in 11-*cis* [2]. The QM/MM trajectories produced in this work were analyzed by Ricardi et al., who developed a novel clustering algorithm to generate average structures of each isomer and use them for excitation energy calculations [31]. They found that the primary structural changes in each of the chromophores were due to conformational changes in both the lysine link and β-ionone ring [31]. The 9-*cis* chromophore was found to have the greatest movement of the lysine chain, and each isomer showed two configurations of the β-ionone ring. The increased movement of the lysine chain is reflected in the average orientation of the 9-*cis* lysine link in comparison to 11-*cis* and all-*trans*, which are roughly the same.

**Figure 4:**
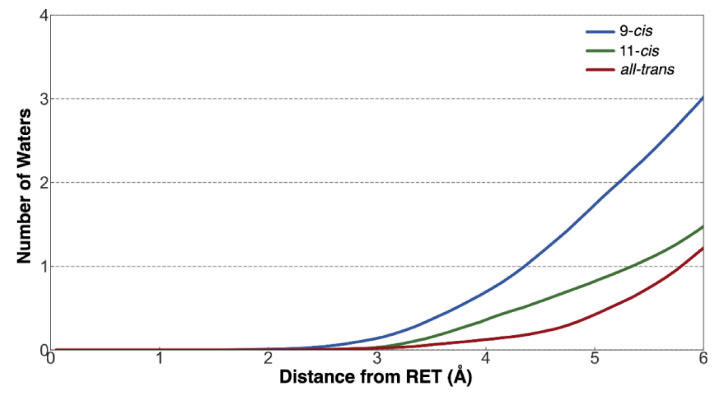
Integrated radial distribution function of waters from the chromophore using the 1-ns QM/MM MD trajectories.

To quantify the amount of water located near the retinal chromophore over the 1-ns QM/MM MD trajectory, the integrated radial distribution function (RDF) was determined for each isomer (Figure 4). The integrated RDF of each isomer shows that, over the course of the QM/MM MD trajectory, there were more waters in close proximity to the 9-*cis*/11-*cis* chromophores than to the all-*trans* isomer, which is consistent with the average structures (Figure 3B–D) [10].

In the all-*trans* model, the methyl functional group pushes water out of the binding pocket and away from the polyene chain due to a twist. Interestingly, the water linking residues E194 and S199 in the 11-*cis* model is found to be positioned slightly further away than that of the 9-*cis* model. The counterion itself is stabilized by residues Y185/Y274, whereas, in the 9-*cis* model, the E194 counterion is stabilized by an additional water found in close proximity to Y185.

### 3.2. Changes in the Electrostatic Environment

**Figure 5:**
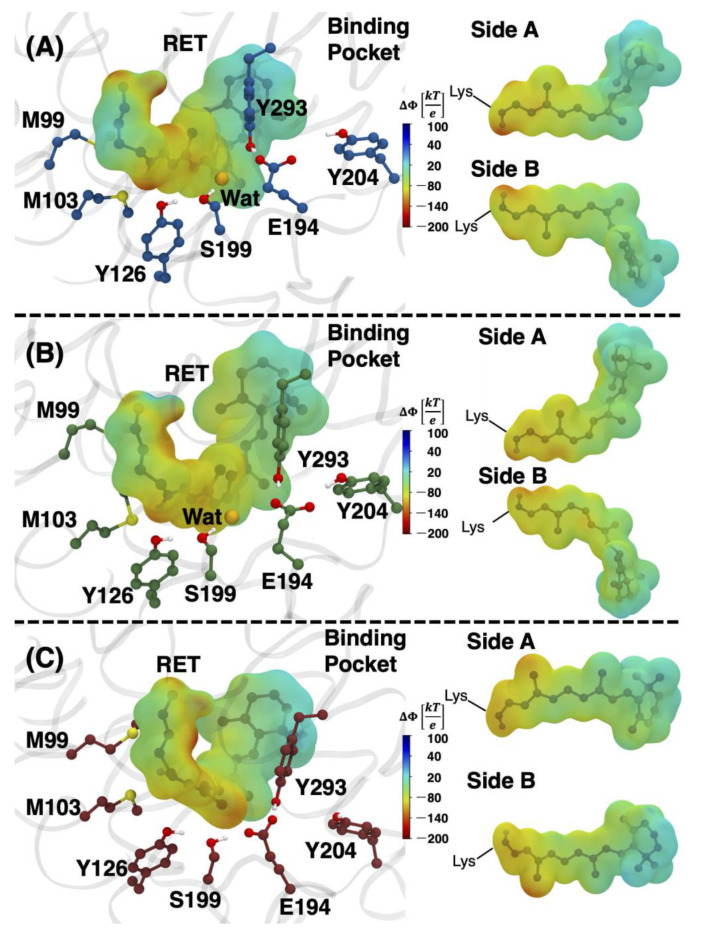
Electrostatic potentials generated for the (**A**) 9-*cis*, (**B**) 11-*cis* and (**C**) all-*trans* models determined using the APBS program and visualized with VMD [32,33]. The electrostatic interactions between the chromophore and the full protein environment were examined. Red indicates a negative electrostatic interaction from the environment, and blue indicates a positive electrostatic interaction. Additionally shown are side views of the retinal chromophore, excluding the lysine side chain, except for the Schiff base nitrogen.

Electrostatic potential maps of the protein environment were generated for the average structure of each isomer model. These maps were then projected onto the van der Waals surface of the corresponding retinal chromophore to visualize the changes of the environment and how it impacts the protein–chromophore interactions (Figure 5). 

The electrostatic potentials show that the 9-*cis* model has a more negative interaction near the RPSB (see the side views of the chromophore in Figure 5A). It appears that the negative electrostatic interaction with Y126 stabilizes the positively charged Schiff base. However, the structural changes of the polyene chain in the all-*trans* model limit the direct interactions of the RPSB with Y126, which instead has a preferential interaction with M99 and M103. This finding is supported by the analysis of the hydrogen bonding partners of the RPSB over the QM/MM MD trajectory. The analysis shows that the Schiff base region of the 9-*cis*, 11-*cis* and all-*trans* models interacted with M103 39.4%, 39.2% and 24.9% of the trajectory, respectively. In addition, the nearby M99 residue is hydrogen-bonded to the Schiff base for 18.3% of the snapshots of all-*trans* but 0% for either *cis* isomer. Both the 9-*cis* and 11-*cis* models interact directly with Y126 for 23.0% and 6.2% of the trajectory, respectively. The E194 counterion does not seem to function as the primary cause of stabilizing interactions in any of the models, because its average distance to the Schiff base, measured as N15 (RET)···O (E194) separation, was 6.95 Å, 6.49 Å and 6.12 Å for the 9-*cis*, 11-*cis* and all-*trans* chromophores, respectively. Therefore, Y126 proximity to the Schiff base appears to play a much larger role in the chromophore–protein interactions, where the average N15 (RET)···O (Y126) distance was 3.04 Å, 3.14 Å and 3.72 Å. This is reflected in the visualization of the electrostatic potentials (Figure 5), which show that the enhanced negative electrostatic interaction in the Schiff base region of the 9-*cis* and 11-*cis* structures occurs near Y126. 

Recently, Bertalan et al. reported MD trajectories obtained with a classical force field and used a graphical analysis of the hydrogen bonding networks inside several proteins and their mutants, including the Iso form of JSR1 [34]. They found that the primary hydrogen bonding partner of the Schiff base over the course of their trajectories was Y126 for 86–90% of the structures studied, with an average distance of 2.9 Å to the Schiff base. Unlike in the crystallographic structure and our QM/MM MD structures, these authors noted that the Schiff base did not bind hydrogen to the M103 residue over the timescale of their trajectories. The discrepancies between the work presented here and that of Bertalan et al. may be explained by differences in the methodology. This might include the use of a different force field or their use of fixed hydrogen bond lengths in the MD simulation, as well as the fact that, in this work, we performed hybrid QM/MM simulations following our long timescale simulation, while they remained in the classical regime.

### 3.3. Absorption Maxima 

**Figure 6:**
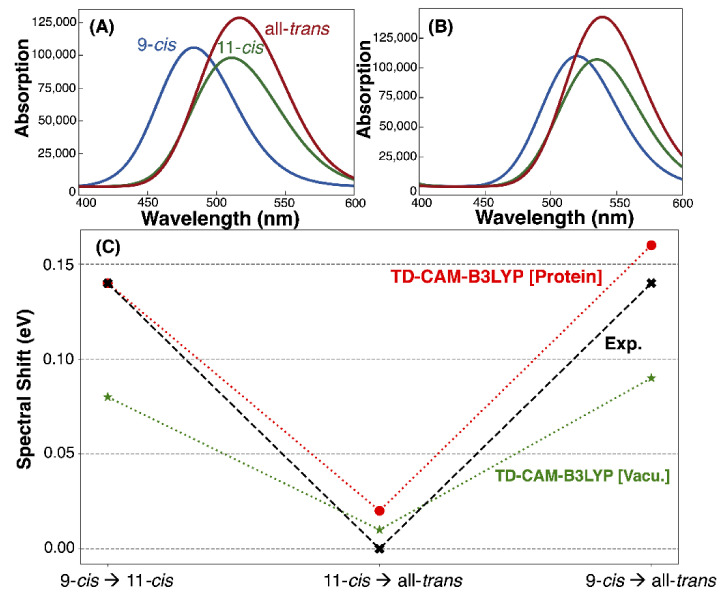
Absorption maxima of the three models determined from 100 snapshots taken from the DFTB2+D trajectories. The excitation energies of these snapshots were calculated using TD-CAM-B3LYP/cc-pVDZ (**A**) in the protein environment and (**B**) in the gas phase. (**C**) The relative shifts between the absorption maxima of the three models for comparison to the experimental shifts from Ehrenberg [2].

The absorption spectra of the isomers were generated from 100 structures taken from the 1-ns QM/MM MD trajectories and using TD-CAM-B3LYP with the cc-pVDZ basis (Figure 6A,B and Table 1). The calculated absorption maxima were 2.56 eV, 2.42 eV and 2.40 eV for the 9-*cis*, 11-*cis* and all-*trans* chromophores, respectively (Figure 6A). The absorption maxima were blue shifted by 0.1 eV relative to their experimental counterpart, well within the normal deviation produced from using TD-DFT methods to calculate vertical excitation energies [35,36,37,38]. The relative shifts between the absorption maxima of each isomer (Figure 6C) were also in good agreement with the experimental values, with an error of 0.02 eV or less. Determining the absorption maximum of each isomer in vacuum (Figure 6B), red shifted the absorption maxima. Both 11-*cis* and all-*trans* were found to be less sensitive to this effect, red shifting by 0.11 eV and 0.10 eV in comparison to 9-*cis*, in which red shifted by 0.18 eV.

**Figure 7:**
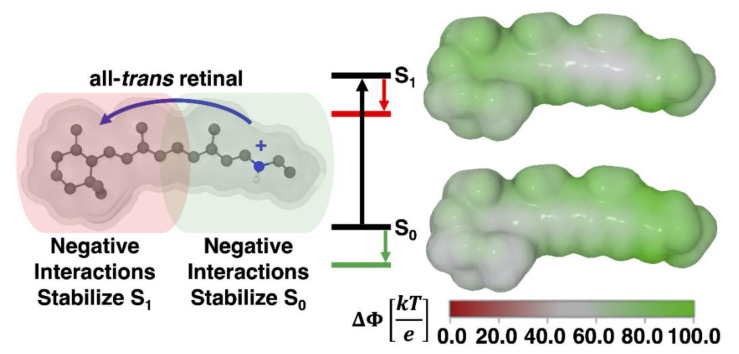
Point charge model schematic for the gas-phase all-*trans* retinal chromophore. Here, the electrostatic potential (ΔΦ) shows a positive charge as green. The schematic shows that the positive charge in the ground state (S_0_) is localized near the Schiff base. Upon excitation to the first excited state (S_1_), the positive charge of the protonated Schiff base moves towards the β-ionone ring.

Spectral tuning in rhodopsins is often explained using the external point charge model developed by Honig and coworkers [39,40,41,42]. This model is based on evidence showing that the S_1_–S_0_ excitation of RPSB is accompanied by a transfer of the positive charge of the Schiff base moiety towards the β-ionone ring (Figure 7). Placing a negatively charged residue at either end of the chromophore can affect the stability of the ground (S_0_) and excited states (S_1_) and, ultimately, create a shift in the absorption maximum.

In our work, the 9-*cis* isomer experienced a larger stabilization of the RPSB by the protein (Figure 6), and thus, embedding this chromophore in the protein environment produced a greater blue shift of the absorption maximum. In comparison, the all-*trans* and 11-*cis* isomers had roughly the same amounts of stabilization of the Schiff base upon being embedded in the protein environment, which helps explain why these models exhibit roughly the same absorption maxima (Table 1) and why moving from vacuum to protein produced a much larger shift for 9-*cis* (0.18 eV) in comparison to 11-*cis* (0.11 eV) and all-*trans* (0.10 eV).

## 4. Conclusions

The spectral shift between the 9-*cis* and 11-*cis*/all-*trans* isomers of the retinal chromophore in JSR1 appear to be controlled by the proximity of a nearby Y126 residue to the RPSB rather than the E194 counterion. The 9-*cis* chromophore is closest to Y126 and has more hydrogen bonding interactions and, in turn, experiences a larger blue shift of the absorption maximum upon inclusion of the protein environment. This is supported by the electrostatic potential maps of each isomer, which shows that the 9-*cis* chromophore has a large negative interaction with the environment near the location of the Y126 residue. Similarly, the distance of the E194 counterion to the Schiff base indicates that it does not appear to have a direct effect on the predicted absorption maxima. There were also key geometric changes found in the models, where the polyene chain of the all-*trans* chromophore twisted in such a way that limited the RPSB from interacting with Y126 and which, instead, interacted with M103/M99 as hydrogen bonding partners. These findings are in agreement with the Raman spectroscopic results of Ehrenberg et al., who noticed that the HOOP modes predicted a different polyene chain orientation between 11-*cis* and all-*trans*. The results of our findings may be used to help explain the stability of JSR1 and other bistable rhodopsins, as well as help guide experimentalists hoping to use these types of proteins as photoswitches for optogenetics.

## Figures and Tables

**Table 1:** Absorption maximum of each isomer in vacuum and in the protein modeled using electrostatic embedding, as well as the experimental values [2,7].

Model	Vacuum (nm, eV)	TD-CAM-B3LYP (nm, eV)	Exp. (nm, eV) [2,7]
9-*cis*	520, 2.38	484, 2.56	505, 2.46
11-*cis*	537, 2.31	512, 2.42	535, 2.32
all-*trans*	540, 2.30	517, 2.40	535, 2.32

## Data Availability

Not applicable.

## Supplementary Materials

The following are available online: Table S1. Constraints (kcal/mol·Å^2^) used during each step of the dynamics with Amber. Table S2. Number of lipid molecules (PA, OL, PC), water (WAT), and ions (Na^+^, Cl^−^) included in the 50 Å QM cutoff region of the end of the QM/MM trajectory. Table S3. RESP fitted atomic point charges from gas-phase trajectories of the retinal chromophores. Also included are the bonding, angle and dihedral information for the 9-*cis*/all-*trans* and 11-*cis*/all-*trans* parameter sets used in the classical simulations with Amber. Figure S1. Visual comparison of the full model to the 50 Å QM cutoff region which was used when determining the excitation energies. Figure S2. Unique atom types used when fitting both the heavy atoms and hydrogens of each isomer of the retinal chromophore.
