# Supplementary material for: The Impact of Retinal Configuration on the Protein–Chromophore Interactions in Bistable Jumping Spider Rhodopsin-1
Source: Molecules. 2021 Dec 23;27(1):71. doi: 10.3390/molecules27010071 (PMC8746357; doi:10.3390/molecules27010071)
Supplement: Supplementary file 1 [file molecules-27-00071-s001.zip › molecules-1486488-supplementary.pdf]

Supplementary Materials

The Impact of Retinal Configuration on the Protein–Chromophore Interactions in Bistable Jumping Spider Rhodopsin-1

Jonathan R. Church[†], Jógvan Magnus Haugaard Olsen[‡] and Igor Schapiro[†,*]

† Fritz Haber Center for Molecular Dynamics Research Institute of Chemistry, The Hebrew University of Jerusalem, Jerusalem 9190401, Israel

‡ DTU Chemistry, Technical University of Denmark, DK-2800 Kongens. Lyngby, Denmark.

* *Corresponding author:* Igor.Schapiro@mail.huji.ac.il

Table of Contents

Equilibration and Production 1

Table S1. Constraints (kcal/mol·Å^2) used during each step of dynamics with Amber. 1

Table S2. Number of lipid molecules (PA, OL, PC), water (WAT), and ions (Na+, Cl-) included in the 50 Å QM cutoff region of the end of the QM/MM trajectory. 2

Figure S1. Visual comparison of the (A) full model to the (B) 50 Å QM cutoff region which was used when determining the excitation energies. 2

Forcefield Parameters 3

Figure S2. Unique atom types used when fitting both the heavy atoms and hydrogens of each isomer of the retinal chromophore. 3

Table S3. RESP fitted atomic point charges from gas-phase trajectories of the retinal chromphores at 300K at the HF/6-31G* level of theory. 3

Bonding, angle and dihedral parameters for 9-*cis*/all-*trans* 5

Bonding, angle and dihedral parameters for 11-*cis*/all-*trans* 12

Equilibration and Production

As mentioned in the main text, the crystal structure of JSR1 (PDB ID: 6I9K) with the all-*trans*, 9-*cis* and 11-*cis* isomers of the retinal chromophore were embedded in a membrane using the CHARMM-GUI website. These systems were minimized, heated and then long time-scale dynamics were performed for 380 ns using the AMBER ff14SB and lipid14 forcefields along with our derived retinal chromophore parameters.[15] The restraints used in each step are outline in Table S1. Each structure was first minimized using the classical force field for 100,000 steps. During the minimization step a restraint weight of 10 kcal/mol·Å² was placed on all heavy and light atoms. A thermal equilibration step was then performed by heating the models from 0 to 300 K stepwise over 50 ps using a time step of 1 fs, followed by constant temperature and volume for the remaining 450 ps. Next, six separate steps of equilibration were performed for 250 ps each at a constant pressure of 1 atm and temperature of 300 K and slowly releasing the constraints on the heavy and light atoms. Following the release of the constraints, a long time-scale classical step was performed for 300 ns at constant pressure and temperature with a 2 fs timestep. Finally, SHAKE was removed from the protein and an additional 80 ns equilibration step was performed with a timestep of 1 fs.

Table S1. Constraints (kcal/mol·Å²) used during each step of dynamics with Amber.

Equilibration step	Protein	Lipid	Ions
**NVT (500 ps)**	10.0	5.0	10.0
**NPT 1 (250 ps)**	10.0	5.0	10.0
**NPT 2 (250 ps)**	5.0	2.5	0.0
**NPT 3 (250 ps)**	2.5	1.0	0.0
**NPT 4 (250 ps)**	1.0	0.5	0.0
**NPT 5 (250 ps)**	0.5	0.1	0.0
**NPT 6 (250 ps)**	0.1	0.0	0.0
**NPT 7 (300 ns)**	0.0	0.0	0.0
**NPT without SHAKE (80 ns)**	0.0	0.0	0.0

The trajectories were then continued as outlined in the main text to determine the absorption maxima of the three isomers.

QM Cutoff Analysis

Table S2. Number of lipid molecules (PA, OL, PC), water (WAT), and ions (Na+, Cl-) included in the 50 Å QM cutoff region of the end of the QM/MM trajectory.

**Lipids**	**9-*cis***	**11-*cis***	**all-*trans***
PA	248	245	245
OL	249	246	247
PC	244	238	241
**Solvent**	**9-*cis***	**11-*cis***	**all-*trans***
Wat	11143	11444	11384
**Ions**	**9-*cis***	**11-*cis***	**all-*trans***
Na+	43	45	37
Cl-	30	39	28

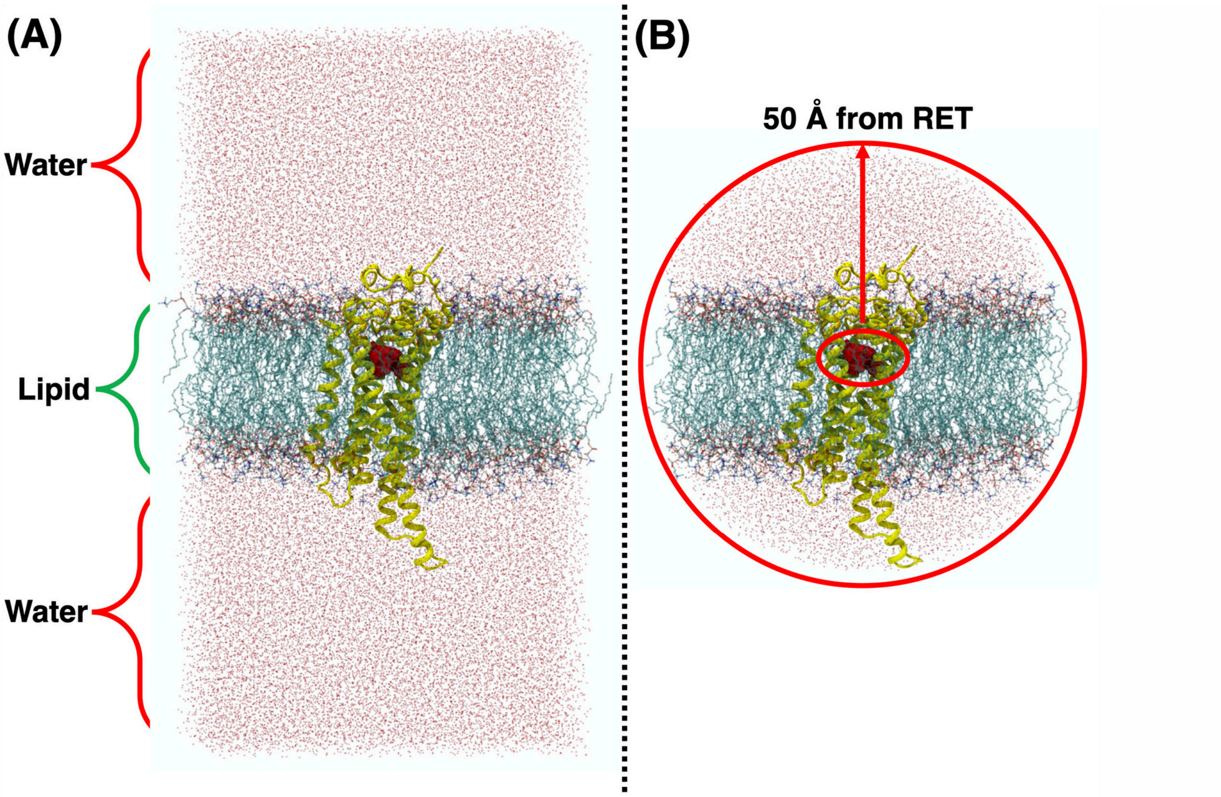

Figure S1. Visual comparison of the **(A)** full model to the **(B)** 50 Å QM cutoff region which was used when determining the excitation energies.

Forcefield Parameters

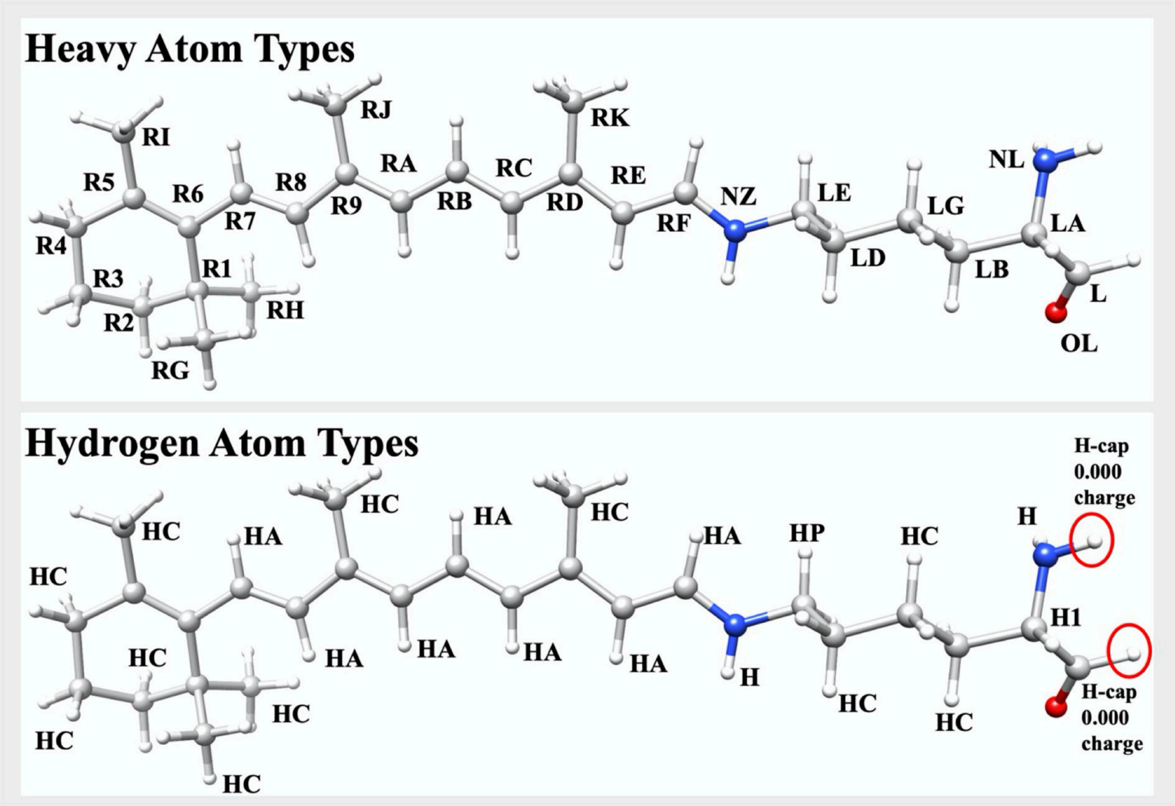

Figure S2. Unique atom types used when fitting both the heavy atoms and hydrogens of each isomer of the retinal chromophore.

RESP Fitted Atomic Point Charges

Table S3. RESP fitted atomic point charges from gas-phase trajectories of the retinal chromphores at 300K at the HF/6-31G* level of theory.

Atom Name	Atom Type	9-cis	11-cis	all-trans
C	L	0.8099	0.8099	0.8099
C1	R1	0.1469	0.1483	0.1469
C10	RA	-0.2994	-0.2298	-0.2994
C11	RB	0.0297	0.0233	0.0297
C12	RC	-0.2497	-0.2698	-0.2497
C13	RD	0.2119	0.2645	0.2119
C14	RE	-0.3766	-0.3275	-0.3766
C15	RF	0.1861	0.0186	0.1861
C16	RG	-0.1813	-0.2479	-0.1813
C17	RH	-0.2537	-0.2239	-0.2537

C18	RI	-0.2086	-0.2330	-0.2086
C19	RJ	-0.2092	-0.1964	-0.2092
C2	R2	-0.0722	-0.0550	-0.0722
C20	RK	-0.1759	-0.1745	-0.1759
C3	R3	-0.1166	-0.1202	-0.1166
C4	R4	-0.078	-0.0823	-0.078
C5	R5	0.1071	0.0972	0.1071
C6	R6	-0.1534	-0.1435	-0.1534
C7	R7	-0.0741	-0.0275	-0.0741
C8	R8	-0.1417	-0.1906	-0.1417
C9	R9	0.1453	0.1334	0.1453
CA	LA	-0.4911	-0.4911	-0.4911
CB	LB	-0.0092	-0.0092	-0.0092
CD	LD	-0.0980	-0.0980	-0.0980
CE	LE	-0.0720	-0.0687	-0.0720
CG	LG	-0.0454	-0.0454	-0.0454
H	H	0.3115	0.3115	0.3115
H10	HA	0.1641	0.1491	0.1641
H11	HA	0.1654	0.1392	0.1654
H12	HA	0.1453	0.1339	0.1453
H14	HA	0.1667	0.1621	0.1667
H15	HA	0.2044	0.0701	0.2044
H161	HC	0.0558	0.0701	0.0558
H162	HC	0.0558	0.0701	0.0558
H163	HC	0.0558	0.0644	0.0558
H171	HC	0.0700	0.0644	0.0700
H172	HC	0.0700	0.0644	0.0700
H173	HC	0.0700	0.0748	0.0700
H181	HC	0.0673	0.0748	0.0673
H182	HC	0.0673	0.0748	0.0673
H183	HC	0.0673	0.0881	0.0673
H191	HC	0.0881	0.0881	0.0881
H192	HC	0.0881	0.0881	0.0881
H193	HC	0.0881	0.0869	0.0881
H201	HC	0.0863	0.0869	0.0863
H202	HC	0.0863	0.0869	0.0863
H203	HC	0.0863	0.2252	0.0863
H21	HC	0.0450	0.04505	0.0450
H22	HC	0.0450	0.04505	0.0450
H31	HC	0.0576	0.05280	0.0576

H32	HC	0.0576	0.05280	0.0576
H41	HC	0.0530	0.05680	0.0530
H42	HC	0.0530	0.05680	0.0530
H7	HA	0.1489	0.1285	0.1489
H8	HA	0.1022	0.1120	0.1022
HA	H1	0.2246	0.2246	0.2246
HB2	HC	0.0680	0.0680	0.0680
HB3	HC	0.0680	0.0680	0.0680
HD2	HC	0.0608	0.0608	0.0608
HD3	HC	0.0608	0.0608	0.0608
HE2	HP	0.1209	0.1252	0.1209
HE3	HP	0.1209	0.1252	0.1209
HG2	HC	0.0514	0.0514	0.0514
HG3	HC	0.0514	0.0514	0.0514
HZ	H	0.3678	0.3931	0.3678
N	N	-0.3661	-0.3661	-0.3661
NZ	NZ	-0.3806	-0.3455	-0.3806
O	OL	-0.6015	-0.6015	-0.6015

Bonding, Angle and Dihedral Parameters

Bonding, angle and dihedral parameters for 9-*cis*/all-*trans*

Generated by mdgx executing mdgx_fitting_bonds.

```
MASS
L  12.01        ! unique type for C
LA 12.01        ! unique type for CA
LB 12.01        ! unique type for CB
LG 12.01        ! unique type for CG
LD 12.01        ! unique type for CD
LE 12.01        ! unique type for CE
NL 14.01        ! unique type for N
NZ 14.01        ! unique type for NZ
OL 16.00        ! unique type for O
R1 12.01        ! unique type for C1
R2 12.01        ! unique type for C2
R3 12.01        ! unique type for C3
R4 12.01        ! unique type for C4
R5 12.01        ! unique type for C5
R6 12.01        ! unique type for C6
R7 12.01        ! unique type for C7
R8 12.01        ! unique type for C8
```

R9 12.01	! unique type for C9
RA 12.01	! unique type for C10
RB 12.01	! unique type for C11
RC 12.01	! unique type for C12
RD 12.01	! unique type for C13
RE 12.01	! unique type for C14
RF 12.01	! unique type for C15
RG 12.01	! unique type for C16
RH 12.01	! unique type for C17
RI 12.01	! unique type for C18
RJ 12.01	! unique type for C19
RK 12.01	! unique type for C20

BOND

NL-H	426.8028	1.0162	Fit by mdgx
L -HC	346.7098	1.0930	Fit by mdgx
LA-H1	338.6221	1.1100	Fit by mdgx
LB-HC	366.8890	1.0758	Fit by mdgx
LG-HC	334.2486	1.1108	Fit by mdgx
LD-HC	338.6117	1.0972	Fit by mdgx
LE-HP	351.9825	1.0890	Fit by mdgx
RF-HA	371.2562	1.0606	Fit by mdgx
RE-HA	371.2206	1.0968	Fit by mdgx
RK-HC	356.2529	1.1100	Fit by mdgx
RC-HA	370.2684	1.0792	Fit by mdgx
RB-HA	369.2150	1.0896	Fit by mdgx
RA-HA	370.6908	1.0967	Fit by mdgx
RJ-HC	354.8543	1.0897	Fit by mdgx
R8-HA	363.8498	1.1009	Fit by mdgx
R7-HA	365.7867	1.0757	Fit by mdgx
RI-HC	325.0520	1.1137	Fit by mdgx
R4-HC	318.2765	1.1246	Fit by mdgx
R3-HC	337.8613	1.0963	Fit by mdgx
R2-HC	342.0748	1.1059	Fit by mdgx
RG-HC	348.8212	1.0843	Fit by mdgx
RH-HC	341.4025	1.0966	Fit by mdgx
NL-LA	324.8670	1.4258	Fit by mdgx
LA-LB	284.7696	1.5290	Fit by mdgx
LA-L	288.4019	1.5347	Fit by mdgx
LB-LG	301.7416	1.5210	Fit by mdgx
LG-LD	295.7298	1.5041	Fit by mdgx
LD-LE	295.2976	1.5349	Fit by mdgx
LE-NZ	339.8687	1.5005	Fit by mdgx
NZ-H	428.8020	0.9817	Fit by mdgx
NZ-RF	490.6085	1.3420	Fit by mdgx
RF-RE	429.0651	1.4122	Fit by mdgx

RE-RD	547.9294	1.3918	Fit by mdgx
RD-RK	299.6053	1.4939	Fit by mdgx
RD-RC	428.4118	1.4309	Fit by mdgx
RC-RB	550.7879	1.3766	Fit by mdgx
RB-RA	428.6174	1.3980	Fit by mdgx
RA-R9	549.2515	1.3563	Fit by mdgx
R9-RJ	319.1907	1.4697	Fit by mdgx
R9-R8	427.6380	1.4283	Fit by mdgx
R8-R7	548.7094	1.3174	Fit by mdgx
R7-R6	429.3807	1.4789	Fit by mdgx
R6-R5	548.3661	1.3115	Fit by mdgx
R6-R1	291.9545	1.5662	Fit by mdgx
R5-RI	314.7533	1.4979	Fit by mdgx
R5-R4	316.7791	1.4852	Fit by mdgx
R4-R3	309.4102	1.5228	Fit by mdgx
R3-R2	308.8226	1.5094	Fit by mdgx
R2-R1	308.4750	1.5033	Fit by mdgx
R1-RG	294.4744	1.5258	Fit by mdgx
R1-RH	296.4695	1.5334	Fit by mdgx
L -OL	591.0309	1.2141	Fit by mdgx

ANGLE

OL-L -HC	64.1832	120.70	Fit by mdgx
NL-LA-H1	130.6212	109.50	Fit by mdgx
H -NL-LA	15.0650	118.04	Fit by mdgx
HC-L -LA	47.7366	109.50	Fit by mdgx
LA-LB-HC	68.4537	109.50	Fit by mdgx
H1-LA-LB	91.3367	109.50	Fit by mdgx
H1-LA-L	57.5827	109.50	Fit by mdgx
LB-LG-HC	79.6901	109.50	Fit by mdgx
HC-LB-HC	31.3902	109.50	Fit by mdgx
HC-LB-LG	76.5499	109.50	Fit by mdgx
LG-LD-HC	68.2410	109.50	Fit by mdgx
HC-LG-HC	35.5596	109.50	Fit by mdgx
HC-LG-LD	82.1086	109.50	Fit by mdgx
LD-LE-HP	45.3307	109.50	Fit by mdgx
HC-LD-HC	35.1436	109.50	Fit by mdgx
HC-LD-LE	85.1538	109.50	Fit by mdgx
HP-LE-HP	40.4953	109.50	Fit by mdgx
HP-LE-NZ	55.8069	109.50	Fit by mdgx
NZ-RF-HA	26.1950	119.10	Fit by mdgx
RF-RE-HA	29.6545	119.70	Fit by mdgx
HA-RF-RE	38.8058	119.70	Fit by mdgx
HA-RE-RD	27.1672	119.70	Fit by mdgx

RD-RK-HC	60.9332	109.50	Fit by mdgx
RD-RC-HA	21.4170	119.70	Fit by mdgx
HC-RK-HC	51.3552	109.50	Fit by mdgx
RC-RB-HA	32.8744	119.70	Fit by mdgx
HA-RC-RB	36.4978	119.70	Fit by mdgx
RB-RA-HA	55.2628	119.70	Fit by mdgx
HA-RB-RA	27.8049	119.70	Fit by mdgx
HA-RA-R9	24.7211	119.70	Fit by mdgx
R9-RJ-HC	32.8361	109.50	Fit by mdgx
R9-R8-HA	33.9935	119.70	Fit by mdgx
HC-RJ-HC	33.8361	109.50	Fit by mdgx
R8-R7-HA	22.4320	119.70	Fit by mdgx
HA-R8-R7	48.0929	119.70	Fit by mdgx
HA-R7-R6	36.7396	119.70	Fit by mdgx
R5-RI-HC	25.5441	109.50	Fit by mdgx
R5-R4-HC	64.3683	109.50	Fit by mdgx
HC-RI-HC	46.3070	109.50	Fit by mdgx
R4-R3-HC	47.9383	109.50	Fit by mdgx
HC-R4-HC	37.7447	109.50	Fit by mdgx
HC-R4-R3	56.9539	109.50	Fit by mdgx
R3-R2-HC	69.0257	109.50	Fit by mdgx
HC-R3-HC	35.1349	109.50	Fit by mdgx
HC-R3-R2	35.4486	109.50	Fit by mdgx
HC-R2-HC	37.3963	109.50	Fit by mdgx
HC-R2-R1	53.4525	109.50	Fit by mdgx
R1-RG-HC	33.1968	109.50	Fit by mdgx
R1-RH-HC	43.0791	109.50	Fit by mdgx
HC-RG-HC	32.5033	109.50	Fit by mdgx
HC-RH-HC	16.4059	109.50	Fit by mdgx
NL-LA-LB	71.5665	109.70	Fit by mdgx
NL-LA-L	96.2895	110.10	Fit by mdgx
LA-LB-LG	50.9188	109.50	Fit by mdgx
LA-L -OL	54.2262	120.40	Fit by mdgx
LB-LA-L	73.3831	111.10	Fit by mdgx
LB-LG-LD	31.7241	109.50	Fit by mdgx
LG-LD-LE	30.1746	109.50	Fit by mdgx
LD-LE-NZ	53.0022	111.20	Fit by mdgx
LE-NZ-H	21.2385	118.40	Fit by mdgx
LE-NZ-RF	37.3790	123.20	Fit by mdgx
NZ-RF-RE	71.6641	122.90	Fit by mdgx
H -NZ-RF	31.1439	120.00	Fit by mdgx
RF-RE-RD	95.2431	122.90	Fit by mdgx
RE-RD-RK	60.2344	119.70	Fit by mdgx
RE-RD-RC	87.5330	122.90	Fit by mdgx
RD-RC-RB	97.5650	122.90	Fit by mdgx
RK-RD-RC	70.0328	119.70	Fit by mdgx

RC-RB-RA	77.2046	122.90	Fit by mdgx
RB-RA-R9	98.3543	122.90	Fit by mdgx
RA-R9-RJ	52.7451	119.70	Fit by mdgx
RA-R9-R8	78.6638	122.90	Fit by mdgx
R9-R8-R7	78.4974	122.90	Fit by mdgx
RJ-R9-R8	70.9083	119.70	Fit by mdgx
R8-R7-R6	64.4190	122.90	Fit by mdgx
R7-R6-R5	86.2291	122.90	Fit by mdgx
R7-R6-R1	48.7179	119.70	Fit by mdgx
R6-R5-RI	44.2240	119.70	Fit by mdgx
R6-R5-R4	71.3832	119.70	Fit by mdgx
R6-R1-R2	57.9885	111.10	Fit by mdgx
R6-R1-RG	84.5453	111.10	Fit by mdgx
R6-R1-RH	66.2120	111.10	Fit by mdgx
R5-R6-R1	62.7417	119.70	Fit by mdgx
R5-R4-R3	68.6735	111.10	Fit by mdgx
RI-R5-R4	43.4471	119.70	Fit by mdgx
R4-R3-R2	34.3569	109.50	Fit by mdgx
R3-R2-R1	52.7358	109.50	Fit by mdgx
R2-R1-RG	55.3001	109.50	Fit by mdgx
R2-R1-RH	81.8735	109.50	Fit by mdgx
RG-R1-RH	68.4933	109.50	Fit by mdgx

DIHEDRAL

HC-L -LA-LB	1	3.02729	0.0	3.0	, Fit by mdgx
HC-L -LA-H1	1	1.92501	180.0	2.0	, Fit by mdgx
NL-LA-L -HC	1	4.53613	180.0	2.0	, Fit by mdgx
NL-LA-LB-HC	1	1.45603	0.0	3.0	, Fit by mdgx
H -NL-LA-H1	1	-6.61343	180.0	2.0	, Fit by mdgx
H -NL-LA-LB	1	-4.72788	180.0	2.0	, Fit by mdgx
H -NL-LA-L	1	-8.32100	180.0	2.0	, Fit by mdgx
LA-LB-LG-HC	1	2.39435	0.0	3.0	, Fit by mdgx
H1-LA-LB-HC	1	-1.88557	0.0	3.0	, Fit by mdgx
H1-LA-LB-LG	1	-0.02323	0.0	3.0	, Fit by mdgx
H1-LA-L -OL	1	9.09531	0.0	-1.0	, Fit by mdgx
H1-LA-L -OL	1	1.22084	180.0	3.0	, Fit by mdgx
LB-LG-LD-HC	1	4.26329	0.0	3.0	, Fit by mdgx
HC-LB-LA-L	1	1.48547	0.0	3.0	, Fit by mdgx
HC-LB-LG-HC	1	-1.57411	0.0	3.0	, Fit by mdgx
HC-LB-LG-LD	1	2.14487	0.0	3.0	, Fit by mdgx
LG-LD-LE-HP	1	-3.11975	0.0	3.0	, Fit by mdgx
HC-LG-LD-HC	1	-2.20890	0.0	3.0	, Fit by mdgx
HC-LG-LD-LE	1	5.82653	0.0	3.0	, Fit by mdgx
HC-LD-LE-HP	1	1.18926	0.0	3.0	, Fit by mdgx
HC-LD-LE-NZ	1	2.51564	0.0	3.0	, Fit by mdgx

LE-NZ-RF-HA	1	6.50151	180.0	2.0	, Fit by mdgx
HP-LE-NZ-H	1	-2.66828	180.0	2.0	, Fit by mdgx
HP-LE-NZ-RF	1	0.78773	180.0	2.0	, Fit by mdgx
NZ-RF-RE-HA	1	4.76512	180.0	2.0	, Fit by mdgx
H -NZ-RF-HA	1	3.77629	180.0	2.0	, Fit by mdgx
HA-RF-RE-HA	1	9.37516	180.0	2.0	, Fit by mdgx
HA-RF-RE-RD	1	6.64781	180.0	2.0	, Fit by mdgx
RE-RD-RK-HC	1	-4.68637	180.0	2.0	, Fit by mdgx
RE-RD-RC-HA	1	0.19686	180.0	2.0	, Fit by mdgx
HA-RE-RD-RK	1	11.71946	180.0	2.0	, Fit by mdgx
HA-RE-RD-RC	1	25.88680	180.0	2.0	, Fit by mdgx
RD-RC-RB-HA	1	11.89246	180.0	2.0	, Fit by mdgx
RK-RD-RC-HA	1	8.09561	180.0	2.0	, Fit by mdgx
HC-RK-RD-RC	1	6.52009	180.0	2.0	, Fit by mdgx
RC-RB-RA-HA	1	11.42848	180.0	2.0	, Fit by mdgx
HA-RC-RB-HA	1	16.98498	180.0	2.0	, Fit by mdgx
HA-RC-RB-RA	1	13.43345	180.0	2.0	, Fit by mdgx
HA-RB-RA-HA	1	-3.30754	180.0	2.0	, Fit by mdgx
HA-RB-RA-R9	1	2.37564	180.0	2.0	, Fit by mdgx
RA-R9-RJ-HC	1	2.46830	180.0	2.0	, Fit by mdgx
RA-R9-R8-HA	1	1.20601	180.0	2.0	, Fit by mdgx
HA-RA-R9-RJ	1	18.53137	180.0	2.0	, Fit by mdgx
HA-RA-R9-R8	1	8.25266	180.0	2.0	, Fit by mdgx
R9-R8-R7-HA	1	29.98571	180.0	2.0	, Fit by mdgx
RJ-R9-R8-HA	1	6.24239	180.0	2.0	, Fit by mdgx
HC-RJ-R9-R8	1	-3.08574	180.0	2.0	, Fit by mdgx
HA-R8-R7-HA	1	18.48239	180.0	2.0	, Fit by mdgx
HA-R8-R7-R6	1	8.88646	180.0	2.0	, Fit by mdgx
HA-R7-R6-R5	1	5.43083	180.0	2.0	, Fit by mdgx
HA-R7-R6-R1	1	-4.09113	180.0	2.0	, Fit by mdgx
R6-R5-RI-HC	1	-3.42140	180.0	2.0	, Fit by mdgx
R6-R5-R4-HC	1	-3.08016	180.0	2.0	, Fit by mdgx
R6-R1-R2-HC	1	-9.53343	0.0	3.0	, Fit by mdgx
R6-R1-RG-HC	1	1.39793	0.0	3.0	, Fit by mdgx
R6-R1-RH-HC	1	-2.36416	0.0	3.0	, Fit by mdgx
R5-R4-R3-HC	1	-5.63222	0.0	3.0	, Fit by mdgx
RI-R5-R4-HC	1	2.67320	180.0	2.0	, Fit by mdgx
HC-RI-R5-R4	1	5.08105	180.0	2.0	, Fit by mdgx
R4-R3-R2-HC	1	3.26166	0.0	3.0	, Fit by mdgx
HC-R4-R3-HC	1	3.90802	0.0	3.0	, Fit by mdgx
HC-R4-R3-R2	1	-7.56303	0.0	3.0	, Fit by mdgx
HC-R3-R2-HC	1	0.99800	0.0	3.0	, Fit by mdgx
HC-R3-R2-R1	1	1.40700	0.0	3.0	, Fit by mdgx
R2-R1-RG-HC	1	-2.25335	0.0	3.0	, Fit by mdgx
R2-R1-RH-HC	1	0.67113	0.0	3.0	, Fit by mdgx
HC-R2-R1-RG	1	2.99226	0.0	3.0	, Fit by mdgx

HC-R2-R1-RH	1	4.30199	0.0	3.0	, Fit by mdgx
RG-R1-RH-HC	1	4.87926	0.0	3.0	, Fit by mdgx
HC-RG-R1-RH	1	1.89917	0.0	3.0	, Fit by mdgx
NL-LA-LB-LG	1	-2.16373	0.0	3.0	, Fit by mdgx
NL-LA-L -OL	1	1.45760	180.0	2.0	, Fit by mdgx
LA-LB-LG-LD	1	3.38161	0.0	-3.0	, Fit by mdgx
LA-LB-LG-LD	1	-0.36767	180.0	-2.0	, Fit by mdgx
LA-LB-LG-LD	1	-3.88540	180.0	1.0	, Fit by mdgx
LB-LA-L -OL	1	3.55249	180.0	2.0	, Fit by mdgx
LB-LG-LD-LE	1	-6.45396	0.0	-3.0	, Fit by mdgx
LB-LG-LD-LE	1	0.55780	180.0	-2.0	, Fit by mdgx
LB-LG-LD-LE	1	4.01621	180.0	1.0	, Fit by mdgx
LG-LB-LA-L	1	0.20369	0.0	3.0	, Fit by mdgx
LG-LD-LE-NZ	1	7.15847	0.0	3.0	, Fit by mdgx
LD-LE-NZ-H	1	-3.35735	180.0	2.0	, Fit by mdgx
LD-LE-NZ-RF	1	1.44495	180.0	2.0	, Fit by mdgx
LE-NZ-RF-RE	1	-2.74556	180.0	2.0	, Fit by mdgx
NZ-RF-RE-RD	1	-2.28184	180.0	2.0	, Fit by mdgx
H -NZ-RF-RE	1	29.51661	180.0	2.0	, Fit by mdgx
RF-RE-RD-RK	1	-4.10868	180.0	2.0	, Fit by mdgx
RF-RE-RD-RC	1	10.82353	180.0	2.0	, Fit by mdgx
RE-RD-RC-RB	1	8.79612	180.0	2.0	, Fit by mdgx
RD-RC-RB-RA	1	-0.67401	180.0	2.0	, Fit by mdgx
RK-RD-RC-RB	1	6.96109	180.0	2.0	, Fit by mdgx
RC-RB-RA-R9	1	1.20673	180.0	2.0	, Fit by mdgx
RB-RA-R9-RJ	1	0.60182	180.0	2.0	, Fit by mdgx
RB-RA-R9-R8	1	-0.48624	180.0	2.0	, Fit by mdgx
RA-R9-R8-R7	1	2.72039	180.0	2.0	, Fit by mdgx
R9-R8-R7-R6	1	1.10481	180.0	2.0	, Fit by mdgx
RJ-R9-R8-R7	1	14.06067	180.0	2.0	, Fit by mdgx
R8-R7-R6-R5	1	-3.45732	180.0	2.0	, Fit by mdgx
R8-R7-R6-R1	1	8.21068	180.0	2.0	, Fit by mdgx
R7-R6-R5-RI	1	14.79333	180.0	2.0	, Fit by mdgx
R7-R6-R5-R4	1	16.99708	180.0	2.0	, Fit by mdgx
R7-R6-R1-R2	1	-9.94111	180.0	2.0	, Fit by mdgx
R7-R6-R1-RG	1	-6.66745	180.0	2.0	, Fit by mdgx
R7-R6-R1-RH	1	-9.70761	180.0	2.0	, Fit by mdgx
R6-R5-R4-R3	1	6.46958	180.0	2.0	, Fit by mdgx
R6-R1-R2-R3	1	11.50829	0.0	3.0	, Fit by mdgx
R5-R6-R1-R2	1	5.70289	180.0	2.0	, Fit by mdgx
R5-R6-R1-RG	1	-4.88921	180.0	2.0	, Fit by mdgx
R5-R6-R1-RH	1	8.55545	180.0	2.0	, Fit by mdgx
R5-R4-R3-R2	1	12.24511	0.0	3.0	, Fit by mdgx
RI-R5-R6-R1	1	12.83161	180.0	2.0	, Fit by mdgx
RI-R5-R4-R3	1	-0.85738	180.0	2.0	, Fit by mdgx
R4-R5-R6-R1	1	4.63806	180.0	2.0	, Fit by mdgx

R4-R3-R2-R1	1	1.99581	0.0	-3.0	, Fit by mdgx
R4-R3-R2-R1	1	-3.43098	180.0	-2.0	, Fit by mdgx
R4-R3-R2-R1	1	-0.43446	180.0	1.0	, Fit by mdgx
R3-R2-R1-RG	1	-7.72084	0.0	-3.0	, Fit by mdgx
R3-R2-R1-RG	1	-4.42902	180.0	-2.0	, Fit by mdgx
R3-R2-R1-RG	1	-5.18926	180.0	1.0	, Fit by mdgx
R3-R2-R1-RH	1	0.00958	0.0	-3.0	, Fit by mdgx
R3-R2-R1-RH	1	-2.14190	180.0	-2.0	, Fit by mdgx
R3-R2-R1-RH	1	1.00932	180.0	1.0	, Fit by mdgx

NONBOND

NL	1.8240	0.1700
L	1.9080	0.0860
OL	1.6612	0.2100
LA	1.9080	0.1094
LB	1.9080	0.1094
LG	1.9080	0.1094
LD	1.9080	0.1094
LE	1.9080	0.1094
NZ	1.9080	0.1094
R1	1.8240	0.1700
R2	1.9080	0.1094
R3	1.9080	0.1094
R4	1.9080	0.1094
R5	1.9080	0.1094
R6	1.9080	0.0860
R7	1.9080	0.0860
R8	1.9080	0.0860
R9	1.9080	0.0860
RA	1.9080	0.0860
RB	1.9080	0.0860
RC	1.9080	0.0860
RD	1.9080	0.0860

Bonding, angle and dihedral parameters for 11-*cis*/all-*trans*

Generated by mdgx executing mdgx_fitting_bonds.

MASS

L 12.01	! unique type for C
LA 12.01	! unique type for CA
LB 12.01	! unique type for CB
LG 12.01	! unique type for CG
LD 12.01	! unique type for CD
LE 12.01	! unique type for CE
NL 14.01	! unique type for N
NZ 14.01	! unique type for NZ

OL 16.00	! unique type for O
R1 12.01	! unique type for C1
R2 12.01	! unique type for C2
R3 12.01	! unique type for C3
R4 12.01	! unique type for C4
R5 12.01	! unique type for C5
R6 12.01	! unique type for C6
R7 12.01	! unique type for C7
R8 12.01	! unique type for C8
R9 12.01	! unique type for C9
RA 12.01	! unique type for C10
RB 12.01	! unique type for C11
RC 12.01	! unique type for C12
RD 12.01	! unique type for C13
RE 12.01	! unique type for C14
RF 12.01	! unique type for C15
RG 12.01	! unique type for C16
RH 12.01	! unique type for C17
RI 12.01	! unique type for C18
RJ 12.01	! unique type for C19
RK 12.01	! unique type for C20

BOND

NL-H	428.6702	1.0040	Fit by mdgx
L -HC	341.7604	1.1071	Fit by mdgx
LA-H1	341.4690	1.0906	Fit by mdgx
LB-HC	369.3077	1.1007	Fit by mdgx
LG-HC	337.3446	1.0951	Fit by mdgx
LD-HC	340.7414	1.0766	Fit by mdgx
LE-HP	350.1632	1.1100	Fit by mdgx
RF-HA	370.5399	1.0721	Fit by mdgx
RE-HA	370.3315	1.0853	Fit by mdgx
RK-HC	344.5658	1.1004	Fit by mdgx
RC-HA	370.4913	1.0905	Fit by mdgx
RB-HA	369.4038	1.0730	Fit by mdgx
RA-HA	370.5350	1.0910	Fit by mdgx
RJ-HC	355.1217	1.1072	Fit by mdgx
R8-HA	363.8549	1.0816	Fit by mdgx
R7-HA	364.4233	1.1070	Fit by mdgx
RI-HC	323.6260	1.0983	Fit by mdgx
R4-HC	319.7262	1.1071	Fit by mdgx
R3-HC	337.4960	1.0938	Fit by mdgx
R2-HC	338.8465	1.1153	Fit by mdgx
RG-HC	347.4206	1.0918	Fit by mdgx
RH-HC	341.5928	1.0949	Fit by mdgx

NL-LA	329.8626	1.4461	Fit by mdgx
LA-LB	288.4904	1.5162	Fit by mdgx
LA-L	285.2538	1.5336	Fit by mdgx
LB-LG	301.2192	1.5168	Fit by mdgx
LG-LD	291.8847	1.5142	Fit by mdgx
LD-LE	292.3313	1.5213	Fit by mdgx
LE-NZ	339.8392	1.4532	Fit by mdgx
NZ-H	428.7219	0.9797	Fit by mdgx
NZ-RF	489.9861	1.3073	Fit by mdgx
RF-RE	428.7718	1.3707	Fit by mdgx
RE-RD	548.6335	1.3744	Fit by mdgx
RD-RK	298.8064	1.4876	Fit by mdgx
RD-RC	427.3170	1.4318	Fit by mdgx
RC-RB	550.6112	1.3596	Fit by mdgx
RB-RA	428.6166	1.3991	Fit by mdgx
RA-R9	549.0277	1.3430	Fit by mdgx
R9-RJ	315.6054	1.4906	Fit by mdgx
R9-R8	427.0305	1.4450	Fit by mdgx
R8-R7	548.1272	1.3443	Fit by mdgx
R7-R6	429.1451	1.4687	Fit by mdgx
R6-R5	545.1223	1.3166	Fit by mdgx
R6-R1	284.8186	1.5531	Fit by mdgx
R5-RI	307.2362	1.4985	Fit by mdgx
R5-R4	315.5420	1.5225	Fit by mdgx
R4-R3	302.0142	1.5146	Fit by mdgx
R3-R2	301.4775	1.5323	Fit by mdgx
R2-R1	308.9021	1.4955	Fit by mdgx
R1-RG	286.7955	1.5504	Fit by mdgx
R1-RH	291.4135	1.5305	Fit by mdgx
L -OL	558.9349	1.2214	Fit by mdgx

ANGLE

OL-L -HC	52.2418	120.70	Fit by mdgx
NL-LA-H1	125.3337	109.50	Fit by mdgx
H -NL-LA	13.0868	118.04	Fit by mdgx
HC-L -LA	40.5239	109.50	Fit by mdgx
LA-LB-HC	64.5874	109.50	Fit by mdgx
H1-LA-LB	95.0440	109.50	Fit by mdgx
H1-LA-L	56.0799	109.50	Fit by mdgx
LB-LG-HC	72.7826	109.50	Fit by mdgx
HC-LB-HC	31.0539	109.50	Fit by mdgx
HC-LB-LG	69.8571	109.50	Fit by mdgx
LG-LD-HC	62.0956	109.50	Fit by mdgx
HC-LG-HC	34.1281	109.50	Fit by mdgx
HC-LG-LD	77.2886	109.50	Fit by mdgx

LD-LE-HP	41.8976	109.50	Fit by mdgx
HC-LD-HC	26.6290	109.50	Fit by mdgx
HC-LD-LE	79.0105	109.50	Fit by mdgx
HP-LE-HP	42.9322	109.50	Fit by mdgx
HP-LE-NZ	47.7374	109.50	Fit by mdgx
NZ-RF-HA	27.4932	119.10	Fit by mdgx
RF-RE-HA	30.3386	119.70	Fit by mdgx
HA-RF-RE	38.3268	119.70	Fit by mdgx
HA-RE-RD	24.7796	119.70	Fit by mdgx
RD-RK-HC	59.5933	109.50	Fit by mdgx
RD-RC-HA	17.2556	119.70	Fit by mdgx
HC-RK-HC	51.4867	109.50	Fit by mdgx
RC-RB-HA	29.8627	119.70	Fit by mdgx
HA-RC-RB	37.6661	119.70	Fit by mdgx
RB-RA-HA	55.8175	119.70	Fit by mdgx
HA-RB-RA	25.4414	119.70	Fit by mdgx
HA-RA-R9	24.3320	119.70	Fit by mdgx
R9-RJ-HC	26.8183	109.50	Fit by mdgx
R9-R8-HA	33.6086	119.70	Fit by mdgx
HC-RJ-HC	30.5184	109.50	Fit by mdgx
R8-R7-HA	24.8913	119.70	Fit by mdgx
HA-R8-R7	49.0561	119.70	Fit by mdgx
HA-R7-R6	26.9002	119.70	Fit by mdgx
R5-RI-HC	21.7455	109.50	Fit by mdgx
R5-R4-HC	63.9883	109.50	Fit by mdgx
HC-RI-HC	41.0156	109.50	Fit by mdgx
R4-R3-HC	48.8829	109.50	Fit by mdgx
HC-R4-HC	38.5348	109.50	Fit by mdgx
HC-R4-R3	46.8087	109.50	Fit by mdgx
R3-R2-HC	68.6090	109.50	Fit by mdgx
HC-R3-HC	29.8070	109.50	Fit by mdgx
HC-R3-R2	32.4812	109.50	Fit by mdgx
HC-R2-HC	36.4995	109.50	Fit by mdgx
HC-R2-R1	49.4804	109.50	Fit by mdgx
R1-RG-HC	31.5374	109.50	Fit by mdgx
R1-RH-HC	45.1968	109.50	Fit by mdgx
HC-RG-HC	23.9768	109.50	Fit by mdgx
HC-RH-HC	12.2239	109.50	Fit by mdgx
NL-LA-LB	74.6856	109.70	Fit by mdgx
NL-LA-L	92.0919	110.10	Fit by mdgx
LA-LB-LG	55.0359	109.50	Fit by mdgx
LA-L -OL	45.1804	120.40	Fit by mdgx
LB-LA-L	73.2216	111.10	Fit by mdgx
LB-LG-LD	36.5207	109.50	Fit by mdgx
LG-LD-LE	46.0239	109.50	Fit by mdgx
LD-LE-NZ	55.6136	111.20	Fit by mdgx

LE-NZ-H	18.7145	118.40	Fit by mdgx
LE-NZ-RF	33.4203	123.20	Fit by mdgx
NZ-RF-RE	70.0010	122.90	Fit by mdgx
H -NZ-RF	31.5362	120.00	Fit by mdgx
RF-RE-RD	92.4287	122.90	Fit by mdgx
RE-RD-RK	62.1041	119.70	Fit by mdgx
RE-RD-RC	79.4094	122.90	Fit by mdgx
RD-RC-RB	87.8023	122.90	Fit by mdgx
RK-RD-RC	67.3628	119.70	Fit by mdgx
RC-RB-RA	68.0012	122.90	Fit by mdgx
RB-RA-R9	97.2480	122.90	Fit by mdgx
RA-R9-RJ	48.4179	119.70	Fit by mdgx
RA-R9-R8	75.5587	122.90	Fit by mdgx
R9-R8-R7	79.8826	122.90	Fit by mdgx
RJ-R9-R8	68.2324	119.70	Fit by mdgx
R8-R7-R6	60.4160	122.90	Fit by mdgx
R7-R6-R5	85.0451	122.90	Fit by mdgx
R7-R6-R1	45.8213	119.70	Fit by mdgx
R6-R5-RI	34.1125	119.70	Fit by mdgx
R6-R5-R4	72.3714	119.70	Fit by mdgx
R6-R1-R2	58.7961	111.10	Fit by mdgx
R6-R1-RG	81.7073	111.10	Fit by mdgx
R6-R1-RH	61.0658	111.10	Fit by mdgx
R5-R6-R1	57.0133	119.70	Fit by mdgx
R5-R4-R3	65.6409	111.10	Fit by mdgx
RI-R5-R4	43.5845	119.70	Fit by mdgx
R4-R3-R2	35.3170	109.50	Fit by mdgx
R3-R2-R1	50.4219	109.50	Fit by mdgx
R2-R1-RG	51.9911	109.50	Fit by mdgx
R2-R1-RH	81.7904	109.50	Fit by mdgx
RG-R1-RH	66.2782	109.50	Fit by mdgx

DIHEDRAL

HC-L -LA-LB	1	5.30468	0.0	3.0	, Fit by mdgx
HC-L -LA-H1	1	0.47809	180.0	2.0	, Fit by mdgx
NL-LA-L -HC	1	5.00808	180.0	2.0	, Fit by mdgx
NL-LA-LB-HC	1	2.12432	0.0	3.0	, Fit by mdgx
H -NL-LA-H1	1	-3.03437	180.0	2.0	, Fit by mdgx
H -NL-LA-LB	1	-1.04762	180.0	2.0	, Fit by mdgx
H -NL-LA-L	1	-6.34236	180.0	2.0	, Fit by mdgx
LA-LB-LG-HC	1	-0.83558	0.0	3.0	, Fit by mdgx
H1-LA-LB-HC	1	-2.17802	0.0	3.0	, Fit by mdgx
H1-LA-LB-LG	1	-1.97635	0.0	3.0	, Fit by mdgx
H1-LA-L -OL	1	0.56338	0.0	-1.0	, Fit by mdgx
H1-LA-L -OL	1	2.03567	180.0	3.0	, Fit by mdgx

LB-LG-LD-HC	1	-1.68500	0.0	3.0	, Fit by mdgx
HC-LB-LA-L	1	-1.13227	0.0	3.0	, Fit by mdgx
HC-LB-LG-HC	1	0.20763	0.0	3.0	, Fit by mdgx
HC-LB-LG-LD	1	0.57494	0.0	3.0	, Fit by mdgx
LG-LD-LE-HP	1	5.37214	0.0	3.0	, Fit by mdgx
HC-LG-LD-HC	1	2.46995	0.0	3.0	, Fit by mdgx
HC-LG-LD-LE	1	-2.06113	0.0	3.0	, Fit by mdgx
HC-LD-LE-HP	1	-1.91318	0.0	3.0	, Fit by mdgx
HC-LD-LE-NZ	1	1.74557	0.0	3.0	, Fit by mdgx
LE-NZ-RF-HA	1	5.65214	180.0	2.0	, Fit by mdgx
HP-LE-NZ-H	1	0.52397	180.0	2.0	, Fit by mdgx
HP-LE-NZ-RF	1	-0.63476	180.0	2.0	, Fit by mdgx
NZ-RF-RE-HA	1	4.92550	180.0	2.0	, Fit by mdgx
H -NZ-RF-HA	1	11.63575	180.0	2.0	, Fit by mdgx
HA-RF-RE-HA	1	0.57584	180.0	2.0	, Fit by mdgx
HA-RF-RE-RD	1	7.15621	180.0	2.0	, Fit by mdgx
RE-RD-RK-HC	1	-1.02775	180.0	2.0	, Fit by mdgx
RE-RD-RC-HA	1	3.05853	180.0	2.0	, Fit by mdgx
HA-RE-RD-RK	1	8.70982	180.0	2.0	, Fit by mdgx
HA-RE-RD-RC	1	8.90268	180.0	2.0	, Fit by mdgx
RD-RC-RB-HA	1	3.93147	180.0	2.0	, Fit by mdgx
RK-RD-RC-HA	1	8.75850	180.0	2.0	, Fit by mdgx
HC-RK-RD-RC	1	3.70049	180.0	2.0	, Fit by mdgx
RC-RB-RA-HA	1	7.77376	180.0	2.0	, Fit by mdgx
HA-RC-RB-HA	1	4.14836	180.0	2.0	, Fit by mdgx
HA-RC-RB-RA	1	4.50722	180.0	2.0	, Fit by mdgx
HA-RB-RA-HA	1	7.00976	180.0	2.0	, Fit by mdgx
HA-RB-RA-R9	1	-2.38774	180.0	2.0	, Fit by mdgx
RA-R9-RJ-HC	1	0.19654	180.0	2.0	, Fit by mdgx
RA-R9-R8-HA	1	-1.74301	180.0	2.0	, Fit by mdgx
HA-RA-R9-RJ	1	3.25857	180.0	2.0	, Fit by mdgx
HA-RA-R9-R8	1	1.20265	180.0	2.0	, Fit by mdgx
R9-R8-R7-HA	1	7.47609	180.0	2.0	, Fit by mdgx
RJ-R9-R8-HA	1	16.80268	180.0	2.0	, Fit by mdgx
HC-RJ-R9-R8	1	0.32290	180.0	2.0	, Fit by mdgx
HA-R8-R7-HA	1	21.69685	180.0	2.0	, Fit by mdgx
HA-R8-R7-R6	1	6.44651	180.0	2.0	, Fit by mdgx
HA-R7-R6-R5	1	0.81358	180.0	2.0	, Fit by mdgx
HA-R7-R6-R1	1	2.19891	180.0	2.0	, Fit by mdgx
R6-R5-RI-HC	1	0.18403	180.0	2.0	, Fit by mdgx
R6-R5-R4-HC	1	-0.61033	180.0	2.0	, Fit by mdgx
R6-R1-R2-HC	1	0.27232	0.0	3.0	, Fit by mdgx
R6-R1-RG-HC	1	-2.32637	0.0	3.0	, Fit by mdgx
R6-R1-RH-HC	1	-0.36726	0.0	3.0	, Fit by mdgx
R5-R4-R3-HC	1	0.42774	0.0	3.0	, Fit by mdgx
RI-R5-R4-HC	1	0.70605	180.0	2.0	, Fit by mdgx

HC-RI-R5-R4	1	2.77513	180.0	2.0	, Fit by mdgx
R4-R3-R2-HC	1	5.04626	0.0	3.0	, Fit by mdgx
HC-R4-R3-HC	1	1.05343	0.0	3.0	, Fit by mdgx
HC-R4-R3-R2	1	-2.09271	0.0	3.0	, Fit by mdgx
HC-R3-R2-HC	1	-0.08517	0.0	3.0	, Fit by mdgx
HC-R3-R2-R1	1	3.98231	0.0	3.0	, Fit by mdgx
R2-R1-RG-HC	1	-0.51018	0.0	3.0	, Fit by mdgx
R2-R1-RH-HC	1	3.14086	0.0	3.0	, Fit by mdgx
HC-R2-R1-RG	1	0.07582	0.0	3.0	, Fit by mdgx
HC-R2-R1-RH	1	-1.51852	0.0	3.0	, Fit by mdgx
RG-R1-RH-HC	1	0.44422	0.0	3.0	, Fit by mdgx
HC-RG-R1-RH	1	3.66769	0.0	3.0	, Fit by mdgx
NL-LA-LB-LG	1	0.95001	0.0	3.0	, Fit by mdgx
NL-LA-L -OL	1	2.73982	180.0	2.0	, Fit by mdgx
LA-LB-LG-LD	1	4.73785	0.0	-3.0	, Fit by mdgx
LA-LB-LG-LD	1	-0.36552	180.0	-2.0	, Fit by mdgx
LA-LB-LG-LD	1	-0.37962	180.0	1.0	, Fit by mdgx
LB-LA-L -OL	1	-0.33867	180.0	2.0	, Fit by mdgx
LB-LG-LD-LE	1	-1.87332	0.0	-3.0	, Fit by mdgx
LB-LG-LD-LE	1	4.72127	180.0	-2.0	, Fit by mdgx
LB-LG-LD-LE	1	-1.47763	180.0	1.0	, Fit by mdgx
LG-LB-LA-L	1	4.03442	0.0	3.0	, Fit by mdgx
LG-LD-LE-NZ	1	-3.22475	0.0	3.0	, Fit by mdgx
LD-LE-NZ-H	1	0.33297	180.0	2.0	, Fit by mdgx
LD-LE-NZ-RF	1	-0.58654	180.0	2.0	, Fit by mdgx
LE-NZ-RF-RE	1	5.96114	180.0	2.0	, Fit by mdgx
NZ-RF-RE-RD	1	1.50332	180.0	2.0	, Fit by mdgx
H -NZ-RF-RE	1	1.52712	180.0	2.0	, Fit by mdgx
RF-RE-RD-RK	1	3.93391	180.0	2.0	, Fit by mdgx
RF-RE-RD-RC	1	-1.45704	180.0	2.0	, Fit by mdgx
RE-RD-RC-RB	1	3.80162	180.0	2.0	, Fit by mdgx
RD-RC-RB-RA	1	5.03410	180.0	2.0	, Fit by mdgx
RK-RD-RC-RB	1	0.98087	180.0	2.0	, Fit by mdgx
RC-RB-RA-R9	1	9.25993	180.0	2.0	, Fit by mdgx
RB-RA-R9-RJ	1	-1.45906	180.0	2.0	, Fit by mdgx
RB-RA-R9-R8	1	6.60943	180.0	2.0	, Fit by mdgx
RA-R9-R8-R7	1	-3.58243	180.0	2.0	, Fit by mdgx
R9-R8-R7-R6	1	5.71745	180.0	2.0	, Fit by mdgx
RJ-R9-R8-R7	1	30.22792	180.0	2.0	, Fit by mdgx
R8-R7-R6-R5	1	1.05014	180.0	2.0	, Fit by mdgx
R8-R7-R6-R1	1	1.05063	180.0	2.0	, Fit by mdgx
R7-R6-R5-RI	1	16.52734	180.0	2.0	, Fit by mdgx
R7-R6-R5-R4	1	4.98574	180.0	2.0	, Fit by mdgx
R7-R6-R1-R2	1	-3.50505	180.0	2.0	, Fit by mdgx
R7-R6-R1-RG	1	1.06578	180.0	2.0	, Fit by mdgx
R7-R6-R1-RH	1	6.43411	180.0	2.0	, Fit by mdgx

R6-R5-R4-R3	1	0.47121	180.0	2.0	, Fit by mdgx
R6-R1-R2-R3	1	6.50165	0.0	3.0	, Fit by mdgx
R5-R6-R1-R2	1	7.81720	180.0	2.0	, Fit by mdgx
R5-R6-R1-RG	1	-6.02495	180.0	2.0	, Fit by mdgx
R5-R6-R1-RH	1	-3.29392	180.0	2.0	, Fit by mdgx
R5-R4-R3-R2	1	3.87747	0.0	3.0	, Fit by mdgx
RI-R5-R6-R1	1	7.28881	180.0	2.0	, Fit by mdgx
RI-R5-R4-R3	1	3.75505	180.0	2.0	, Fit by mdgx
R4-R5-R6-R1	1	15.27044	180.0	2.0	, Fit by mdgx
R4-R3-R2-R1	1	-4.52500	0.0	-3.0	, Fit by mdgx
R4-R3-R2-R1	1	9.38420	180.0	-2.0	, Fit by mdgx
R4-R3-R2-R1	1	4.42482	180.0	1.0	, Fit by mdgx
R3-R2-R1-RG	1	-1.34098	0.0	-3.0	, Fit by mdgx
R3-R2-R1-RG	1	0.02159	180.0	-2.0	, Fit by mdgx
R3-R2-R1-RG	1	0.00101	180.0	1.0	, Fit by mdgx
R3-R2-R1-RH	1	6.03687	0.0	-3.0	, Fit by mdgx
R3-R2-R1-RH	1	-6.18516	180.0	-2.0	, Fit by mdgx
R3-R2-R1-RH	1	4.72302	180.0	1.0	, Fit by mdgx

NONBOND

NL	1.8240	0.1700
L	1.9080	0.0860
OL	1.6612	0.2100
LA	1.9080	0.1094
LB	1.9080	0.1094
LG	1.9080	0.1094
LD	1.9080	0.1094
LE	1.9080	0.1094
NZ	1.9080	0.1094
R1	1.8240	0.1700
R2	1.9080	0.1094
R3	1.9080	0.1094
R4	1.9080	0.1094
R5	1.9080	0.1094
R6	1.9080	0.0860
R7	1.9080	0.0860
R8	1.9080	0.0860
R9	1.9080	0.0860
RA	1.9080	0.0860
RB	1.9080	0.0860
RC	1.9080	0.0860
RD	1.9080	0.0860
